# Analysis of DnaK Expression from a Strain of *Mycoplasma fermentans* in Infected HCT116 Human Colon Carcinoma Cells

**DOI:** 10.3390/ijms22083885

**Published:** 2021-04-09

**Authors:** Sabrina Curreli, Hervé Tettelin, Francesca Benedetti, Selvi Krishnan, Fiorenza Cocchi, Marvin Reitz, Robert C. Gallo, Davide Zella

**Affiliations:** 1 Institute of Human Virology, University of Maryland School of Medicine, Baltimore, MD 21201, USA; FBenedetti@ihv.umaryland.edu (F.B.); SKrishnan@ihv.umaryland.edu (S.K.); FCocchi@ihv.umaryland.edu (F.C.); MReitz@ihv.umaryland.edu (M.R.); RGallo@ihv.umaryland.edu (R.C.G.); 2 Department of Medicine, University of Maryland School of Medicine, Baltimore, MD 21201, USA; 3 Institute for Genome Sciences, Department of Microbiology and Immunology, University of Maryland School of Medicine, Baltimore, MD 21201, USA; tettelin@som.umaryland.edu; 4 Department of Microbiology and Immunology, University of Maryland School of Medicine, Baltimore, MD 21201, USA; 5 Department of Biochemistry and Molecular Biology, University of Maryland School of Medicine, Baltimore, MD 21201, USA

**Keywords:** *Mycoplasma fermentans*, DnaK expression, mRNA, DnaK protein, intracellular localization

## Abstract

Several species of mycoplasmas, including *Mycoplasma fermentans*, are associated with certain human cancers. We previously isolated and characterized in our laboratory a strain of human mycoplasma *M. fermentans* subtype *incognitus* (MF-I1) able to induce lymphoma in a Severe Combined Immuno-Deficient (SCID) mouse model, and we demonstrated that its chaperone protein, DnaK, binds and reduces functions of human poly-ADP ribose polymerase-1 (PARP1) and ubiquitin carboxyl-terminal hydrolase protein-10 (USP10), which are required for efficient DNA repair and proper p53 activities, respectively. We also showed that other bacteria associated with human cancers (including *Mycoplasma* *pneumoniae*, *Helicobacter* *pylori*, *Fusobacterium* *nucleatum*, *Chlamydia* *thrachomatis*, and *Chlamydia pneumoniae*) have closely related DnaK proteins, indicating a potential common mechanism of cellular transformation. Here, we quantify *dnaK* mRNA copy number by RT-qPCR analysis in different cellular compartments following intracellular MF-I1 infection of HCT116 human colon carcinoma cells. DnaK protein expression in infected cells was also detected and quantified by Western blot. The amount of viable intracellular mycoplasma reached a steady state after an initial phase of growth and was mostly localized in the cytoplasm of the invaded cells, while we detected a logarithmically increased number of viable extracellular bacteria. Our data indicate that, after invasion, MF-I1 is able to establish a chronic intracellular infection. Extracellular replication was more efficient while MF-I1 cultured in cell-free axenic medium showed a markedly reduced growth rate. We also identified modifications of important regulatory regions and heterogeneous lengths of *dnaK* mRNA transcripts isolated from intracellular and extracellular MF-I1. Both characteristics were less evident in *dnaK* mRNA transcripts isolated from MF-I1 grown in cell-free axenic media. Taken together, our data indicate that MF-I1, after establishing a chronic infection in eukaryotic cells, accumulates different forms of *dnaK* with efficient RNA turnover.

## 1. Introduction

The role of certain bacteria associated with human cancers in promoting cell transformation is poorly understood except in the case of *H. pylori*, where a causative relationship with stomach cancer has been observed [1], potentially triggered by reducing p53 activities [2]. *M. fermentans* (MF) is a bacterium of the Mollicutes class, which lack a cell wall and have a reduced genome size [3]. Though most mycoplasmas are extracellular, some invade eukaryotic cells [4,5,6,7,8], colonizing different intracellular compartments [9]. The bacteria subsequently enter a chronic infectious stage, where reduced proliferation and the production of a small number of viable particles are observed [10,11,12,13]. Some data indicate that mycoplasmas may facilitate tumorigenesis in humans and in some cases may be directly involved in one or more stages of their cause [14]. In this regard, in vivo infection has been associated with some human cancers, including HIV-seropositive subjects with non-Hodgkin’s lymphoma (NHL) [15], prostate cancer [16], and oral cell carcinoma [17]. In addition, in vitro infection of *M. fermentans* subtype *incognitus* induces chromosomal alterations in both human prostate and murine embryonic cell lines, with the acquisition of malignant properties in mouse and human cells, including loss of anchorage dependency, ability to form colonies in soft agar and tumorigenicity in nude mice [18,19,20]. Finally, several mycoplasmas (*M. fermentans*, *M. arginini*, *M. hominis*, and *M. arthritidis*) inhibit p53 activity and cooperate with Ras in oncogenic transformation, though the responsible bacterial protein was not identified [21].

DnaK is a member of the human Hsp70 chaperone family and a central component of the bacterial chaperone system [22]. DnaK binds to exposed regions of unfolded or partially folded proteins, ensuring that they achieve their proper functional conformation, and it participates in the assembly of large multi-protein complexes, preventing the formation and precipitation of unstable protein aggregates [23]. We previously demonstrated that DnaK from a strain of *M. fermentans* subtype *incognitus* isolated in our laboratory (MF-I1) from an HIV patient binds to human proteins Poly (ADP-Ribose) polymerase 1 (PARP1), central for sensing DNA damage and initiating repair activities, and Ubiquitin carboxyl-terminal hydrolase 10 (USP10) [24], which promotes activity and stabilization of p53, critical for its anti-cancer functions [25]. We than showed that, upon transfection and expression in human adenocarcinoma cells (HCT116), DnaK reduced PARP-dependent DNA repair activities and p53-dependent anti-cancer responses [24,26]. Phylogenetic amino acid analysis shows that other bacteria associated with human cancers (including certain mycoplasmas, *H. pylori*, *F. nucleatum*, and *C. thrachomatis*) have highly related DnaK proteins, suggesting a possible common mechanism of cellular transformation [24,26].

Bacterial proteins, including DnaK, can be translocated into eukaryotic cells either upon attachment to the cellular membrane or upon invasion [27,28]. Thus, it is presumed that DnaK from MF-I1 isolate translocated into cellular compartments could lead to disruption of important cellular pathways. DnaK is known to be expressed by actively proliferating bacteria, and its expression is increased under stress conditions [29,30,31,32]. However, little is known about DnaK expression and regulation by mycoplasma upon cellular invasion.

In this study, we quantified DnaK copy number and expression (mRNA) in different cellular compartments following invasion of HCT116 human colorectal carcinoma cells with MF-I1 strain. We also measured the number and replicative ability of viable intracellular bacteria (assessed by CFU assays), as compared to cell-free bacteria. Circularized reverse transcription coupled with PCR (cRT-PCR) and followed by analysis of cloned and sequenced amplified products was used to compare important regulatory regions and heterogeneous lengths of *dnaK* mRNA transcripts isolated from intracellular mycoplasma and from cell-free particles. We demonstrate that MF-I1 invasion of the eukaryotic cells leads to their chronic infection by viable bacteria with poor replicative capacity, but which still express DnaK. Upon its secretion and accumulation over time in chronically infected cells, this would result in its binding to human proteins, eventually interfering with and reducing the activities of some proteins, as we already demonstrated in the case of PARP1 and p53 [24,26].

## 2. Results

### 2.1. Infection of Human HCT116 Cancer Cells Results in Reduced M. fermentans MF-I1 Replication, Increased Expression of dnaK DNA Copy Number and Detection of Intracellular DnaK

Little is known about the levels of expression and intracellular localization of Mycoplasma *dnaK* during the invasion of eukaryotic cells. We investigated the ability of MF-I1 isolate to invade mammalian cells (HCT116 colon carcinoma) by using a Gentamicin protection assay [33]. Adherent cells were harvested at day 1, day 3, and day 6 following infection by trypsinization followed by multiple PBS washes and qPCR was used to measure intracellular MF-I1 DNA *dnaK* copy numbers over time in culture. Subsequently, expression of *dnaK* was quantified in different cell compartments by qRT-PCR and correlated with viable bacteria recovered intracellularly and quantified by CFU analysis. For comparison, viable bacteria of MF-I1 were also quantified in culture supernatants of the same cells (extracellular) and in MF-I1 cultured in McCoy’s 5A medium (cell-free, axenic medium). Western Blot analysis was used to confirm and quantify expression of DnaK protein in infected cells.

**Figure 1:**
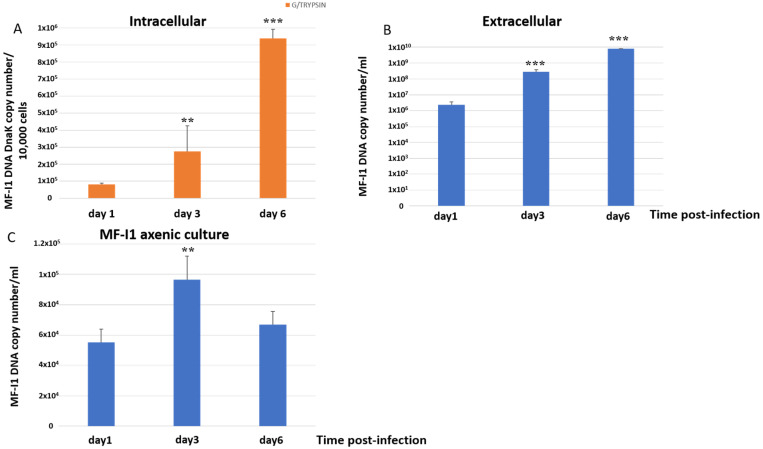
Quantification of *dnaK* DNA copy number. DNA copy number per 10,000 cells infected trypsinized from MF-I1 infected HCT116 cells (**A**) and DNA copy number per ml of supernatant from HCT116 infected cells (**B**) was collected and analyzed at day 1, 3, and 6 after infection. (**C**) DNA from parallel cultures of MF-I1 isolate grown in McCoy’s 5A medium, cell-free, axenic medium was collected and analyzed after 1, 3, and 6 days of culture post-inoculum. MF-I1 DNA copy number was ascertained by Q-PCR using primers specific for the *dnaK* gene. MF-I1 DNA levels in the infected cells were normalized to albumin DNA. Data represent the mean values ± S.E. of samples run in triplicate and are representative of data from three different experiments. The *p*-values were calculated by two sample *t*-test. ** = *p*-value between 0.05 and 0.01; *** = *p*-value < 0.01.

Intracellular MF-I1 DNA copies per 10,000 detached trypsinized cells increased 3.4-fold from day 1 to day 3 post-infection and 11.5-fold by 6 days, reaching a copy number of 9.38 × 10^5^ ± 6.19 × 10^4^/10,000 cells (Figure 1A). The changes were significant (*p* = 0.003 and *p* = 0.0008, respectively). The amount of MF-I1 in culture supernatants increased 119.5-fold (Figure 1B) from day 1 to day 3 of infection (*p* < 0.0001) and 3329 times after 6 days of infection (*p* = 0.0002), reaching a copy number of 7.69 × 10^9^ ± 3.07 × 10^8^/mL. In contrast, when MF-I1 was grown in McCoy’s 5A medium without cells, we observed only a 1.75-fold increase of MF-I1 from day 1 to day 3, and only 1.21-fold (*p* = 0.1) by day 6, reaching a copy number of 6.69 × 10^4^ ± 8.7 × 10^3^/mL (Figure 1C). Thus, the amount of MF-I1 isolate grown axenically on day 6 was 100,000 fold lower than that in the supernatant derived from the culture with HCT116 cells, suggesting that the presence of eukaryotic cells provides critical replication factor/s that improve bacterial growth (compare Figure 1B,C).

We next quantified the viable MF-I1 bacteria in the same three culture conditions (Table 1). We observed a 7.6 fold increase in internalized MF-I1 after 3 days of infection (*p* = 0.002), which decreased to 3.2 fold at day 6 (Table 1). These data suggest that the peak of active growth of MF-I1 inside the cells was reached 3 days post-infection and that the increase in *dnaK* copy number detected by q-PCR represented not only replicating MF-I1, but also defective or non-replicating MF-I1.

**Figure 2:**
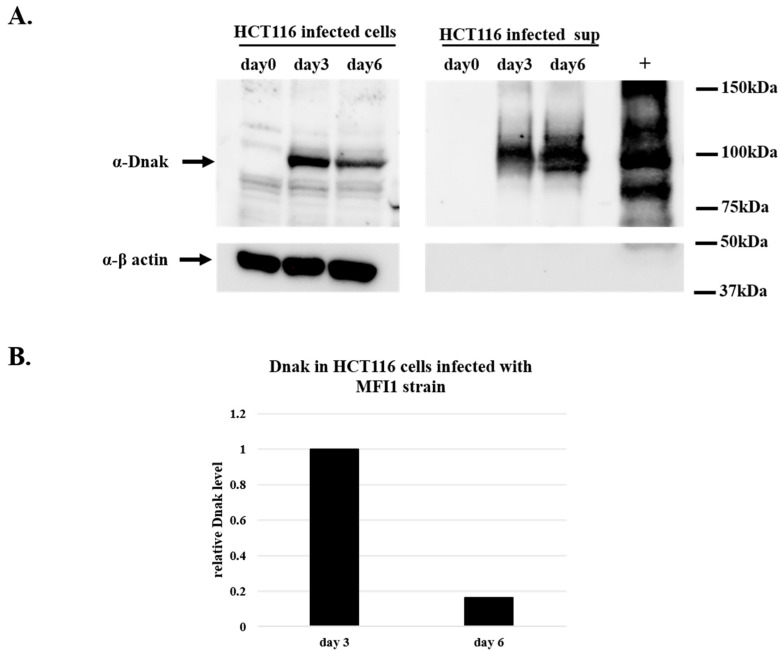
Western blot analysis of DnaK protein in lysates from HCT116 cells infected with MF-I1. Cells were infected with MF-I1 and at day 0, day 3, and day 6 cells and corresponding supernatants were harvested. To ensure the recovery of intracellular Mycoplasma and removal of extracellular mycoplasma from the cells, HCT116 infected cells were treated with gentamicin 400 μg/mL and 0.005% Triton x-100 two hours after removal of the supernatant before harvesting the cells. (**A**) Cellular proteins and cleared supernatants were separated by SDS-PAGE and analyzed by Western blot using anti-DnaK and anti-β actin antibodies. (**B**) The relative amount of DnaK protein in HCT116 cells was calculated by using ImageJ software (NIH, Bethesda).

To demonstrate the synthesis of DnaK protein, HCT116 cells infected with MF-I1 isolate were collected at day 3 and 6 after the infection together with their corresponding supernatants and were analyzed by Western blot using an antibody specific for bacterial DnaK (see Material and Methods). The presence of DnaK was observed at day 3 and 6 after the infection, both inside the cells and in the supernatants (Figure 2). The amount of recovered protein intracellularly was four times higher at day 3 of the infection compared to the amount of proteins recovered after 6 days of infection (Figure 2B). This is in agreement with the results from the colony assay (Table 1), indicating that the peak of infection in HCT116 cells is reached at day 3. In contrast, the amount of protein observed in the cell’s supernatant increased with time (Figure 2A).

In agreement with q-PCR and Western blot, CFUs/mL of MF-I1 in the supernatants from infected HCT116 cells increased 4.1 fold (Table 1) from day 1 to day 3 post-infection and 41.1-fold 6 days post-infection. In contrast, the amount of bacteria grown axenically in McCoy’s 5A medium after 6 days of culture (in CFUs/mL) was 3-fold less than the amount of MF-I1 grown in the supernatant derived from the HCT116 cell cultures (Table 1). Specifically, the CFU/mL of MF-I1 grown axenically increased only 3.9 fold from day 1 to day 3 of infection and 8.7 times after 6 days of infection, and these increases were not significant (*p* = 0.1 and *p* = 0.6, respectively). Our data indicate that the isolated MF-I1 *M. fermentans* strain reaches a steady state replication and number of viable bacteria inside the cells, as previously observed with other bacteria species [10,11,12,13]. Our data also indicate that MF-I1 replication rate is more efficient in the supernatant of eukaryotic cells compared to the axenic culture, likely due in part to nutrient factors (e.g., glycerol metabolites and extracellular DNA) released by the cells [34,35]. Although MF-I1 survived in axenic culture, it only did so with a significantly reduced replication rate.

**Figure 3:**
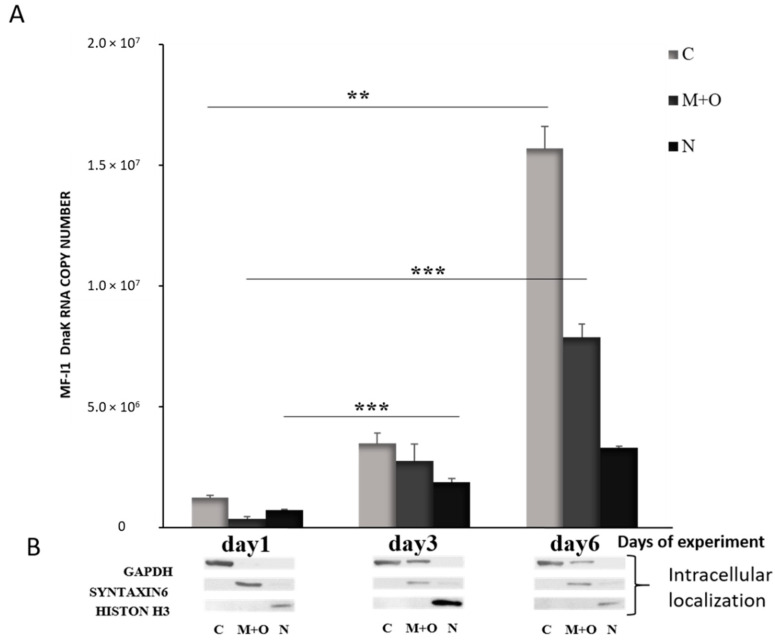
Quantification of *dnaK* RNA copy number in different intracellular compartments of HCT116 infected with *M. fermentans* MF-I1. (**A**): After the indicated days of experiment, RNA was collected from the different cellular compartments, and subjected to qRT-PCR analysis. (**B**): Western blot was performed to confirm proper fractionation of cell compartments. GAPDH was used as a marker for cytosol, Syntaxin 6 was used to label the membrane compartment and organelles and H3 for the nucleus. Data represent the mean values ± S.E. of samples run in triplicate and are representative of data from three different experiments. The *p*-values were calculated by two sample *t*-test. ** = *p*-value between 0.05 and 0.01; *** = *p*-value < 0.01. Cellular compartments are indicated as follows: C; Cytoplasm; M + O: Mitochondria + organelles; N: Nucleus.

When we analyzed the expression of *dnaK* RNA in different cellular compartments, we detected increased expression over time, and the majority of RNA was found in the cytoplasm of the cells by day 6 (Figure 3). DNA qPCR analysis confirmed that MF-I1 was indeed mostly localized in the cytoplasm (Figure S1). Interestingly, by comparing these data with the quantification of viable bacteria (compare Figure 1C and Table 1), it is clear that while MF-I1 reached a steady of state level inside the cells, the amount of *dnaK* RNA was clearly still increasing, indicating active continuous expression.

### 2.2. Analysis of MF-I1 dnaK Transcripts Exposes the Emergence of Bacteria with Several Mutations in Important Regulatory Regions

To characterize the termini of the *dnaK* transcripts and identify their positions with respect to *dnaK* adjacent genes *rnhB1* (located 5′), encoding ribonuclease H, and *mgs1* (located 3′), encoding an ATPase, we performed circularized RT-PCR (cRT-PCR) (Figure S2A) using a set of primers (Table S1) designed to specifically amplify by nested PCR both ends of the RNAs, which were subsequently cloned and sequenced. Since mycoplasmas are self-replicating microorganisms that often infect eukaryotic cells, we decided to compare three in vitro systems, an axenic MF-I1 culture grown in 243 media (the same medium used to produce MF-I1 stocks), MF-I1 grown in HCT116 cultures chronically infected with MF-I1, and an MF-I1 control grown in McCoy’s 5A medium without HCT116 cells. Table S3A,B show DNA sequences of 12 clones of *dnaK* termini derived from MF-I1 axenic cultures in 243 medium (Table S3A) and from HCT116 cells infected with MF-I1 for 6 days (Table S3B). Additionally, in infected HCT116 cultures the 5′ termini of *dnaK* transcripts were variable and ranged from −50 to −30 nucleotides (nt) upstream from the ATG translation start codon. We also detected some shorter clones that lacked the ATG codon. In contrast, only in one clone out of 12 derived from MF-I1 axenic cultures in 243 medium did we detect a *dnaK* 5′ terminus (Table S3A,B). No *dnaK* 5′ or 3′-end sequences were detected from 12 clones analyzed from a parallel MF-I1 axenic culture in McCoy’s 5A medium. Absence of detectable *dnaK* terminal sequences in MF-I1 axenic cultures in McCoy’s 5A medium suggests that in the lack of eukaryotic cells McCoy’s medium does not support MF-I1 replication and that the number of analyzed clones may have been too limited for detecting transcripts containing *dnaK* 5′ and 3′-ends.

As one of five heat shock-induced genes, mycoplasma *dnaK* contains a regulatory CIRCE element [30,36], which negatively regulates *dnaK* expression upon interaction with the heat-inducible transcription repressor HrcA (heat regulation at CIRCE), a mechanism that is widespread in bacteria [37]. Using the online program http://emboss.bioinformatics.nl/cgi-bin/emboss/palindrome (URL accessed on 14 January 2019), we searched for potential CIRCE elements in the upstream region of the *dnaK* gene of *M. fermentans*. We found an inverted CIRCE-like repeat sequence (TTAGCACT-N_10_-GAGTGCTAA) spanning from nt −75 to −50 upstream the *dnaK* gene of *M. fermentans* strain JER (CP001995) and a similar sequence (TTAGCACT-N_11_-GAGTGCTAA) at nt −76 to −50 from the *dnaK* gene of *M. fermentans* strain MF-I1 (ATFG00000000). Another palindrome-like sequence (AAAAAATAATTA-N_70_-TAATTATTTTTT) spanning from nt −75 to −144 upstream *dnaK* gene was identified in both *M. fermentans* strains. It is of future interest to determine whether both inverted regions interact with the HrcA protein.

The sequences at the 3′-end of *dnaK* mRNA (Table S3A,B) show a common poly-T termination sequence (TTTTTTAT) +37 to +44 nt downstream from the TAG stop signal (the positions were identical for *M. fermentans* MF-I1 and *M. fermentans* JER). Analysis with an online software program for transcription terminators in bacterial genomes (http://www.softberry.com/berry.phtml?topic=findterm&group=programs&subgroup=gfindb, URL accessed on 14 January 2019) predicted a Rho-independent transcription terminator oriented 3′ to 5′, starting +29 nt downstream from the TAG stop signal and with the length of 42 nt [38]. Most of the clones analyzed from HCT116 cells infected with *M. fermentans* strain MF-I1 retained the poly-T sequence, while 7 clones of 12 from axenic cultures in 243 medium were shorter and lacked the poly-T termination sequence, but retained the CIRCE element.

Similar to *dnaK*, the sequences in the *rnhB1* 5′-end termini (Table S4A,B) displayed variable lengths, with shorter 5′ -ends ranging from −20 to −55 nt and longer ones ranging from −100 to −200 nt upstream from the ATG starting codon. One *M. fermentans* clone from the HCT116 supernatants had 5′ ends at −299. In contrast, sequences in the *rnhB1* 5′-end terminal from *M. fermentans* MF-I1 axenic culture in McCoy’s 5A medium displayed only shorter 5′ -ends ranging from −29 to −55 nt (Table S4C). Like *dnaK*, *rnhB1* 3′-end had a common poly-T termination sequence (TTATTTTTTT) in position +85 to +88 nt downstream from the TAG stop signal (Table S5A–C). However, 8 clones out of 12 analyzed from McCoy’s 5A medium axenic cultures in Table S5C completely lacked the termination sequence. Therefore, similarly to *dnaK*, *rnhB1* mRNAs appear to be monocystronic when analyzed from either axenic cultures or from HCT116 cells. However, the 5′ terminal region includes the end of the adjacent gene, tagged as MFE_01010 in *M. fermentans* JER (CP001995), which encodes the COF family HAD hydrolase protein.

Compared to *dnaK* and *rnhB1* end transcripts, we obtained a different result from analyzing *mgs1* end transcripts (Tables S6 and S7A,B). In most clones the RNA started with an ATG start codon (Table S6). However, some clones lacked the origin, suggesting that *mgs1* mRNAs can start with either the ATG or from alternative internal promoters. The *msg1* 3′-end of clones derived from *M. fermentans* MF-I1 axenic cultures in 243 medium contained nucleotide sequences ranging from +124 nt to +64 nt (Table S7A), while clones derived from HCT116 cells infected with *M. fermentans* MF-I1 contained both longer sequences spanning from +422 nt to +135 nt and shorter nucleotide sequences ranging from +90 nt to + 51 nt (Table S7B). One clone derived from infected HCT116 cells was extremely long (+1361 nt). The long mRNAs included sequences from the adjacent *pheS* gene, encoding for the phenylalanyl-tRNA synthetase alpha subunit, as well as from part of the following *ung* gene, encoding uracil-DNA glycosylase, and suggesting that mgs1 together with *pheS* and *ung* can be transcribed as multicistronic mRNAs (Table S7B). Only 2 shorter nucleotide sequences ranging from +82 nt to + 75 nt were detected in *msg1* 3′-end clones derived from *M. fermentans* MF-I1 axenic cultures in McCoy’s 5A medium (Table S7C)*,* while the remaining 10 clones lacked the *msg1* 3′-end.

Polyadenylation degradation intermediates were not detected in *dnaK* RNAs, nor in the *dnaK* -adjacent genes *rnhB1* and *mgs1*, in agreement with a previous report for *M. gallisepticum* [39], suggesting that Mycoplasma mRNA is not polyadenylated.

### 2.3. M. fermentans MF-I1 Strain dnaK mRNA Length Analysis Reveals Presence of Several Types of Transcripts

**Figure 4:**
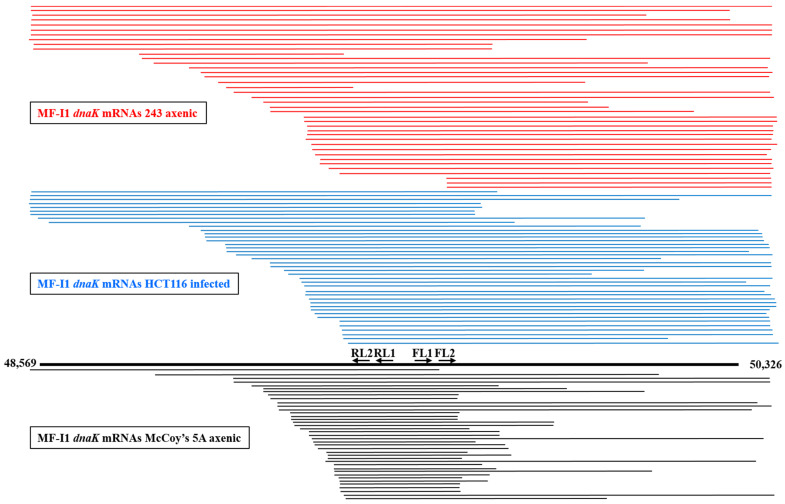
Schematic representation of *dnaK* RNA length obtained by cRT-PCR analysis. Mycoplasma *dnaK* mRNA lengths are indicated. The *dnaK* gene with the ATG start and TAA terminal codons is represented by the thick black line in the bottom. Primer pairs FL1/RL1 and FL2/RL2 used for cRT-PCR are also represented. Horizontal lines representing the cDNAs obtained by cRT-PCR technique are drawn to scale. The top lines in red represent the sequences derived from *dnaK* RNAs from the MF-I1 axenic culture in 243 medium, middle lines in blue represent sequences derived from *dnaK* RNAs from HCT116 cells infected with MF-I1, and the bottom lines in black represent the sequences derived from *dnaK* RNAs from the MF-I1 axenic culture in McCoy’s 5A medium. Numbers indicate the nucleotide position of the clone sequences, according to the sequence in GenBank accession (ATFG00000000) comprising the complete *M. fermentans* MF-I1 genome.

Bacteria express different species of RNA transcripts from a single gene [40], and our finding of some *dnaK* mRNAs clones lacking the ATG start codon at the 5′-end (Table S3) prompted us to look further at *dnaK* mRNA expression. We therefore analyzed *dnaK* mRNA sizes by cRT-PCR. However, in contrast with the previous 5′ and 3′-termini analyses, specific primers were selected to be complementary to the middle region of the RNA transcripts (Figure S2B and Table S2). A total of 121 clones were analyzed, including 40 clones derived from MF-I1 axenic cultures in 243 medium, 41 from MF-I1-infected HCT116 cells, and 40 from MF-I1 axenic cultures in McCoy’s 5A medium. Figure 4 shows a schematic representation of the obtained sequences, and Figure S3 a multiple sequence alignment.

Sequence alignments revealed extensive length variation compared to the expected size of MF-I1 *dnaK* mRNA. Specifically, the 3′ terminus was frequently complete, including the terminal hairpin stretch (+1404) in 25 sequences (out of 41) derived from infected HCT116 cells and 24 sequences from the MF-I1 cell-free culture in 243 (out of 40) (Figure 4 and Figure S3), while only 6 sequences contained the hairpin stretch in MF-I1 axenic growth in McCoy’s 5A medium. Surprisingly, most of the sequences were truncated at the 5′-end. Only 5 sequences, including 4 originating from the MF-I1 axenic culture in 243 medium and 1 from infected HCT116 cells, consisted of mature full-length transcripts that started between −37 and −30 nt before the ATG start codon (therefore including the *dnaK* promoter region), in agreement with the data from the *dnaK* 5′-end analysis (Table S3A,B). No complete sequence was detected in MF-I1 axenic cultures in McCoy’s 5A medium. Six clones from MF-I1 cell-free cultures in 243, 7 from infected HCT116 cells, and 1 from MF-I1 axenic cultures in McCoy’s 5A medium included sequences upstream from the ATG start codon, but were truncated at the 3′-end.

All remaining sequences, including 33 derived from HCT116 cells infected with MF-I1, 30 from MF-I1 axenic 243 cultures, and 39 from MF-I1 axenic cultures in McCoy’s 5A medium were truncated at the 5′-end. In particular, the transcripts were highly heterogeneous, but could be aligned in a staircase-like pattern. However, while the remaining 30 sequences derived from MF-I1 axenic cultures in 243 medium (including those beginning from +218 and +772 nt downstream from the ATG start codon) showed a staircase-like pattern, the 33 mRNA sequences derived from HCT116 cells infected with MF-I1 showed a staircase-like pattern that included shorter sequences beginning from +407 and +783 nt after the ATG start codon. The sequences with a staircase-like pattern were even shorter in the 39 mRNA sequences derived from MF-I1 axenic culture in McCoy’s 5A medium, beginning from +517 and +782 nt after the ATG start codon. Additionally, most of the mRNAs derived from MF-I1 axenic culture in McCoy’s 5A medium (34 clones out of 40) were truncated at the 3′-end, especially at positions between +1035 and +1183 nt after the ATG start codon, while fewer mRNA sequences derived from both MF-I1 cell-free 243 culture and HCT116 cells infected with MF-I1 (16 clones for both) were randomly truncated at the 3′-end (Figure 4 and Figure S3).

These newly identified 5′-end truncated *dnaK* mRNA sequences are likely the result of a 5′-end RNA decay process, previously observed in Gram-positive and -negative bacteria [41,42]. To this regard, we observed that 5′-end truncated *dnaK* mRNAs were similarly enriched in MF-I1 cell-free 243 culture and in infected HCT116, while both 5′ and 3′-end *dnaK* mRNAs were exceedingly truncated in MF-I1 axenic cultures in McCoy’s 5A medium. This would likely indicate that MF-I1 RNA turnover is more rapid and efficient during infection of eukaryotic cells and axenic growth in nutrient-rich 243 medium than during free bacterial growth in McCoy’s 5A medium.

### 2.4. Bacteria Grown in Different Conditions Show Differences in dnaK mRNA and Its Internal Promoter

To find out whether 5′-end truncated *dnaK* mRNA sequences represented nascent mRNAs originating from the alternative internal promoters, we looked for *dnaK* internal promoter/s. Using the web server http://www.softberry.com/berry.phtml?topic=bprom&group=programs&subgroup=gfindb (URL accessed on 14 January 2019), we identified four putative dnaK promoters, shown in Table 2. Promoter 1 spanned from nt −173 to −145 upstream from the ATG start codon of *dnaK*. This promoter was also upstream from the inverted CIRCE-like repeat sequence located from nt −76 to −50 upstream from the *dnaK* gene of MF-I1, as reported above in this paper. The three remaining potential promoters were located within the *dnaK* gene. Promoter 2 spanned from nt +197 to +227 downstream from the ATG start codon of *dnaK* and promoter 3 spanned from nt +617 to +647. Promoter 4 was quite far from the ATG start codon of *dnaK* (Table 2) and only 552 nt distant from the *dnaK* stop codon.

We next used the Blast program (https://blast.ncbi.nlm.nih.gov/Blast.cgi, URL accessed on 14 January 2019) [43] to verify that some 5′-end truncated *dnaK* mRNAs likely originate from the newly identified potential promoters. We found that promoters 2 and 3 were frequently included in *dnaK* transcripts, but none of these transcripts identified with cRT-PCR were long enough at their 5′-end to include promoter 1. Only one transcript from axenic MF-I1 cultures in 243 medium (C25) contained promoter 2 at the origin of the 5′-end truncated *dnaK* mRNA sequence, although in various sequences promoter 2 was present internally. In contrast, 6 sequences from MF-I1 axenic cultures in McCoy’s 5A medium (M3, M8, M12, M29, M31, and M33) and 2 sequences (H13 and H37) from HCT116 chronically infected with MF-I1 for 6 days had promoter 3 localized at the origin of the 5′ -end truncated *dnaK* mRNA sequence. We could not identify promoter 4 in *dnaK* transcripts because the cRT-PCR amplicons enriched for *dnaK* mRNAs were limited to the region spanning from nt +1036 to the ATG start codon (Table S2). These results suggest that some of the 5′-end truncated *dnaK* transcripts originate from internal promoters; however, further studies will be necessary to prove the use of the putative *dnaK* internal promoters in *M. fermentans* MF-I1.

Analysis of the mRNA sequences from HCT116 cells infected with MF-I1, MF-I1 axenic culture in 243 medium, and MF-I1 axenic culture in McCoy’s 5A medium revealed that 41% and 35% of *dnaK* mRNA transcripts from HCT116 infected and MF-I1 axenic cultures in 243 media, respectively, carried mutations, while only 12% of *dnaK* mRNA transcripts from MF-I1 axenic culture in McCoy’s 5A medium were mutated (Table 3). The majority of the mutated sequences carried single point mutations, with the exception of two frame shift mutations consisting of one deletion in a sequence from HCT116 cells infected with MF-I1 and one insertion in a sequence from an MF-I1 axenic culture in 243 medium. Of the single point mutations, 22% from HCT116 infected cultures, 21% from MF-I1 axenic cultures in 243 medium, and 50% from MF-I1 axenic cultures in McCoy’s 5A medium were synonymous. The remaining single point mutations led to amino acid changes, and in one case resulted in a stop signal. A common single point mutation, consisting of the substitution of A with G at +1190 nt after the ATG start codon, leading to the substitution of Glu with Gly in position 397 of the *dnaK* protein, was detected in four sequences from HCT116 infected with *M. fermentans* MF-I1.

## 3. Materials and Methods

### 3.1. Culture of Bacteria

*M. fermentans* subtype *incognitus* (MF-I1) was isolated in 2014 at the Institute of Human Virology from HIV-1 infected cell supernatants [24] and grown in 243 medium containing heart infusion broth (BD Biosciences, San Jose, CA, USA) supplemented with 20% heat inactivated horse serum and 10% yeast extract solution (ThermoFisher Scientific, Waltham, MA, USA), at 37 °C in aerobic conditions. MF-I1 cultures were harvested in late log phase, collected by centrifugation (30 min at 10,000× *g* at 4 °C), and washed three times with PBS before use. Cells were infected with a concentration of 2 CFU (colony forming units)/cell. More details about *M. fermentans* MF-I1, including data deposition information, were described previously [24].

### 3.2. Colony Forming Unit Assay

Bacterial inoculum was serially diluted in 243 bacterial media derived from ATCC, and 50 μL of the dilution was plated in duplicate in 60 × 15 mm culture dishes with 2 × 2 mm grid culture dishes with 2 × 2 mm grid and 21.5 cm^2^ surface (Fisher Scientific, Hampton, NH, USA). After 7 days routine manual counting was performed by two independent individuals for plates with less than 200 colonies under an inverted microscope. Colony Forming Units (CFU)/mL were determined by multiplying the average number of colonies/grids × number of grids in the plate (53,750) × the dilution factor × 20 (for 50 μL of inoculum).

### 3.3. Cells and MF-I1 Infection

The human cancer cell line HCT116 was obtained from American Type Culture Collection (ATCC CCL-243^TM,^ Manassas, VA, USA). Cells were maintained in McCoy’s 5A medium, supplemented with 10% fetal bovine serum, 50 IU penicillin, 50 µg/mL streptomycin, and L-glutamine (290 µg/mL) (all from ThermoFisher Scientific, Waltham, MA, USA). For time point experiments, HCT116 cells infected with MF-I1 were harvested at day 1, 3, and 6. Parallel cultures of uninfected HCT116 were the negative control. To avoid cell overgrowth in the late time points, flasks of HCT116 cells were split at a 1/4 ratio after 24h and at a 1/6 ratio after 72h. To remove extracellular MF-I1, cells were treated with gentamicin 400 μg/mL and 0.005% Triton x-100 (both from Sigma-Aldrich, St. Louis, MO, USA) 2 h before harvesting the cells, as previously described [33]. Cell monolayers were washed twice in PBS, trypsinized, and washed 3 times in PBS to remove extracellular MF-I1. Cell culture supernatants were first centrifuged at 400× *g* to remove residual cells, and MF-I1 was then collected by centrifugation (30 min at 10,000× *g* at 4 °C). Parallel control cultures of MF-I1 growing in McCoy’s 5A medium without HCT116 cells were harvested at the same time points.

### 3.4. Accession Numbers

Nucleotide numbering of *rnhB1*, *dnaK*, and *mgs1* RNAs is according to NCBI GenBank accession no, ATFG00000000 defining the complete genome of *M. fermentans* MF-I1. *M. fermentans* JER (accession no CP001995) was used for multiple alignment analyses.

### 3.5. DNA and RNA Extraction

Cellular DNA was extracted using PrepEase Genomic DNA Isolation Kits (USB Corporation, Cleveland, OH, USA), following instruction from the manufacturer. MF-I1 DNA from cellular supernatants was extracted using PureLink Viral RNA/DNA Mini Kit (ThermoFisher Scientific, Waltham, MA, USA). For RNA analyses, pellets from control MF-I1 axenic cultures grown overnight in 243 medium or for six days in McCoy’s 5A medium without HCT116 cells were lysed in TRIzol (ThermoFisher Scientific, Waltham, MA, USA). RNA was extracted with chloroform, precipitated in isopropanol (both from Sigma-Aldrich, St. Louis, MO, USA), suspended in DEPC water, and used for cRT-PCR experiments. Similarly, HCT116 cultures infected with MF-I1 and uninfected controls were maintained in culture for 6 days and then harvested for RNA analyses using the TRIzol protocol.

### 3.6. Analysis of Subcellular Fractions by SDS-PAGE and Immunoblot

Subcellular fractionation was performed using the Qproteome cell compartment kit from Qiagen (#37502, Qiagen, Hilden, Germany) and following the instructions from the manufacturer. For immunoblot analysis cytosol, membranes and nuclear compartments were isolated with the specific extraction buffer from the kit. After precipitation with acetone, the dry pellets were resuspended in RIPA buffer (ThermoFisher Scientific, Waltham, MA, USA) containing a protease inhibitor mixture (Sigma-Aldrich). 30 µg of each lysate were resolved by electrophoresis on a 16% SDS-polyacrylamide gel using Tris-glycine-SDS running buffer (ThermoFisher Scientific, Waltham, MA, USA), electrotransferred to PVDF membrane (Bio-Rad, Hercules, CA, USA), by using Trans-Blot Turbo system (Bio-Rad, Hercules, CA, USA) and membranes were probed with antibodies specific for each fraction (all from Cell Signaling Technology, Danvers, MA, USA). Specifically, rabbit mAb anti-glyceraldehyde-3-phosphate dehydrogenase (GAPDH) (cat. N 5174) was used as a marker for cytosol, rabbit mAb anti Syntaxin 6 (cat. N 2869) was used to label the membrane compartment and mouse mAb anti-Histone H3 (cat. N 3638) for the nucleus. RNA and DNA extractions from subcellular fractions were performed by using the TRIzol protocol (ThermoFisher Scientific, Waltham, MA, USA) or PrepEase Genomic DNA Isolation Kits (USB Corporation, Cleveland, OH, USA), respectively.

For Western blot analysis of DnaK protein, 70 µg of cell lysate and 10 µg of clarified supernatants were resolved by electrophoresis. PVDF membranes containing the transferred proteins were probed with anti-DnaK mouse mAb (Novus Biological, Littleton, CO, USA) and anti–β-actin rabbit mAb (Cell Signaling Technology, Danvers, MA, USA) antibodies. Blots were first incubated with a secondary HRP-conjugated antibody (Cell Signaling), then developed using an ECL chemiluminescent substrate kit (Genesee Scientific, San Diego, CA, USA), finally exposed and acquired using the ChemiDoc MP digital image system (Bio-Rad, Hercules, CA, USA).

### 3.7. Cloning of MF-I1 dnaK

The nucleotide sequence of *dnaK* was obtained from the MF-I1 genomic sequence (Genbank accession number: ATFG00000000) and, along with a 5′ overhang of four nucleotides (CACC) to facilitate directional cloning, was synthesized (Blue Heron Biotechnology, Bothell, WA) and cloned into the pcDNA 3.1 Directional TOPO expression vector (ThermoFisher Scientific, Waltham, MA, USA) according to the manufacturer’s recommendations.

### 3.8. Quantitative PCR (qPCR) for MF-I1 and Host Cell Genome Copy Numbers

10^7^ copies of pcDNA 3.1-*dnaK* plasmid were diluted in a 1:10 serial dilution five times in 40 ng of uninfected HCT116 DNA and used as a standard template. The absolute copy numbers of MF-I1 in 40 ng DNA derived from infected cells were determined by comparison with a standard curve. For relative qPCR, intracellular MF-I1 isolate copy numbers were normalized to host genome copy numbers. HCT116 genomic DNA was extracted from uninfected cells and 100 ng of DNA, corresponding to 20,000 cells, was diluted in a 1:10 serial dilution and utilized as standard template. The albumin gene (GenBank accession number: EF649953.1) was used as reference gene to normalize input genomic DNAs. qPCR was performed using an iQ5 thermocycler Real-Time PCR Detection System with an iQ SYBR Green Supermix (both from Bio-Rad, Hercules, CA, USA). The reaction conditions were: 15 s at 95 °C followed by 35 cycles of denaturation for 15 s at 95 °C and annealing and extension for 60 s at 60 °C. Primers for the MF-I1 *dnaK* gene were: Forward, 5′-CCTGATGAAGTTGTTGCAATGG-3′ and reverse, 5′-ACCAAGTGTAAGAGGTGTAACG-3′. Primers for host cell albumin were: forward, 5′-GCTGTCATCTCTTGTGGGCTGT-3′ and reverse, 5′-ACTCATGGGAGC TGCTGGTTC-3′. The MF-I1 isolate copy number in 10,000 cells was determined from the ratio of MF-I1 copy number/(albumin copy number × 2) × 10,000 (the 2-fold factor is because the albumin sequence is present at two copies per genome).

### 3.9. Circularized RT-PCR (cRT-PCR) for Analysis of 5′ and 3′ Ends of MF-I1 Isolate mRNA

Analysis of 5′ and 3′ -ends of *M. fermentans* MF-I1 strain RNAs was as described previously [44] with minor changes. In brief, the 5′ cap was removed using RppH (New England Biolabs Inc., Ipswich, MA, USA) [45] in NEB Thermopol buffer (1× = 20 mM Tris-HCl, 10 mM (NH4)2SO4, 10 mM KCl, 2 mM MgSO4, 0.1% Triton^®^ X-100, pH 8.8) for 1 h at 37 °C. Decapped RNA was extracted with phenol/chloroform and precipitated with 3M NaOAc, pH 5.5, and 2.5 volumes of ethanol. Circularization of 5 μg of RNA was performed at 37 °C for 1 hr using T4 RNA ligase (Life Technologies Corporation, Grand Island, NY, USA), DNAse 1 (New England Biolabs Inc., Ipswich, MA, USA), and RNase inhibitor. C-RNA was extracted with phenol/chloroform and precipitated with 3M NaOAc, pH 5.5, and 2.5 volumes of ethanol. Reverse transcription of circularized RNA was performed using primers R1 (see Figure S2 and Table S1) proximal to the origin of the MF-I1 RNA for *dnaK* or the adjacent genes *rnhB1* and *mgs1*. Reverse transcription was performed for 1 h at 55 °C in 25 μL using Superscript^TM^ III Reverse Transcriptase with DTT 0.1M, dNTPs 10mM, and RNaseOut (all from ThermoFisher Scientific, Waltham, MA, USA). Enzymes were inactivated at 70 °C (15 min). 2.5 μL of the cRT reaction were used for the sequential PCR 1 reaction, with gene specific primers R2 (internal to R1 primer used for the RT reaction), and F1 proximal to the end of the gene (Table S1). PCR used AccuPrime^TM^ Taq DNA Polymerase High Fidelity (ThermoFisher Scientific, Waltham, MA, USA) with an initial denaturation at 94 °C for 3 min. cDNA was denatured at 94 °C for 30 s, annealed at 58 °C for 30 s, and elongated at 68 °C for 1 min. An elongation step of 10 min followed the 30 cycles of PCR. The PCR product was purified from a 1% agarose gel (ThermoFisher Scientific, Waltham, MA, USA) using the Qiagen gel purification Kit and eluted in 15 μL vol. water. A second nested PCR was performed to guarantee the specificity of the reaction by using internal primers R2 and F2 (Table S1). The same protocol used for PCR 1 was used for PCR 2 reactions and the products purified in 1% agarose. Purified PCR 2 product was eluted in 15 μL volume and A-Tailed for 20 min at 70 °C with Taq DNA Polymerase and 0.2 mM dATP (both from ThermoFisher Scientific, Waltham, MA, USA). The product was cloned into the T/A pGEM-T Easy Vector (Promega Corporation, Madison, WI, USA) following the manufacturer’s instructions. Clones were subsequently selected and grown overnight. Plasmid DNA was isolated using Qiagen mini-prep kits and the insert sequenced by the Biopolymer/Genomics Core Facility at the University of Maryland, School of Medicine.

To analyze *dnaK* mRNA length, circularized RT-PCR was performed using the same protocol used for analyses of 5′ and 3′-ends of MF-I1 isolate mRNA. However, the *dnaK* primers were positioned in the center of the *dnaK* gene (see Figure S2 and Table S2).

### 3.10. Sequence Analyses

We used the RNIE [46] program in sensitive mode to predict hairpins in *MF-I1* isolate *dnaK* RNA and the web server: http://www.softberry.com/berry.phtml?topic=bprom&group =programs&subgroup=gfindb (accessed on 14 January 2019) to predict internal promoters in *MF-I1 dnaK* DNA. Multiple sequence alignments were created using the MUSCLE program [47]. The consensus included a sequence logo in order to help visualize/identify rare mutations [48].

Statistical analyses. Statistical analyses used GraphPad Prism v 5.0. For categorical independent variables, differences between groups were assessed using the Student’s *t*-test.

## 4. Discussion

Although mycoplasmas are generally considered extracellular pathogens, some species, including *M. fermentans*, can invade human cells and grow intracellularly [4,5,6,7]. In this regard, *M. fermentans* has been shown to fuse, invade, and reside in intracellular vacuoles [9,49]. Invasion of host cells may contribute to mycoplasma persistence, providing protection from the host immune response and antibiotic treatment, while at the same time releasing bacterial proteins inside the cell. We previously demonstrated that a chaperone protein, DnaK, from a *M. fermentans* subtype *incognitus* (MF-I1) isolated in our laboratory, binds and reduces functions of human poly-ADP ribose polymerase-1 (PARP1) and ubiquitin carboxyl-terminal hydrolase protein-10 (USP10), which are required for efficient DNA repair and proper p53 activities, respectively. Other bacteria associated with human cancers (*M. pneumoniae*, *H. pylori*, *F. nucleatum, C. thrachomatis*, and *C. pneumoniae*) have closely related DnaK proteins, indicating a potential common mechanism of cellular transformation [24]. DnaK is the major bacterial Heat Shock Protein 70 (Hsp70) [50] and is one of the most abundant constitutively expressed and stress inducible chaperones in bacteria [22]. As a molecular chaperone, it prevents protein misfolding and aggregation, ensuring proteome integrity [50]. The expression of bacterial heat shock genes is efficiently controlled at the transcriptional level by both positive [51] and negative mechanisms [37]. However, little is known about the levels and localization of *dnaK* RNA expression in bacterial particles upon cellular invasion, especially during the reduced levels of bacterial replication leading to chronic infection. By measuring intracellular viable bacterial particles and correlating them with *dnaK* RNA and DnaK protein expression, we demonstrated that expression of *dnaK* is continuous and elevated even after MF-I1 reached a steady-state levels inside the cells. In contrast, the amount of extracellular viable bacteria increased logarithmically, likely due in part to nutrient factors released by the host cells [34,35]. Further confirming this hypothesis, MF-I1 growth in cell-free, in axenic culture showed a significantly reduced replication rate.

By analyzing *dnaK* RNA using cRT-PCR and sequencing we demonstrated that the gene is expressed as a monocistronic mRNA, and identified the regulatory element CIRCE in the 5′ region and a Rho-independent hairpin terminator in the 3′ region. In addition, multiple sequence alignments revealed extensive variation in MF-I1 *dnaK* transcript length compared to the expected mRNA size. Notably, while the 3′-terminus was frequently complete and included the terminal hairpin region, most of the transcripts were truncated at the 5′-end. In particular, the alignment of *dnaK* transcripts presented a staircase-like pattern. Based on our data and the literature [40,52,53], it is likely that 5′-end truncated *dnaK* mRNA sequences result from an RNase-mediated degradation process. RNA decay is a critical mechanism for regulating gene expression in bacteria. Bacterial mRNA is degraded rapidly, with an average half-life of 2.0 min or less [54], and our analysis of RNA length likely showed multiple intermediates of this process. We hypothesize that decay is more frequent at the 5′-end because the 3′-end is protected by hairpin termination.

Bacteria have evolved complex mechanisms to survive under conditions of limited nutrients, and these responses affect gene expression and regulation [55,56,57]. Bacteria growth rates vary depending upon the type or amount of available nutrients, and many other growth-rate dependent parameters (such as gene and plasmid copy numbers, the abundance of RNA polymerases and ribosomes) also affect gene expression [58]. However, while the gene copy number per bacteria is growth rate dependent [59], the degradation rate of mRNA appears to be rather independent of growth rate [52], and in *E. coli* is attributed to autoregulation of RNase E [53]. Regulation of *dnaK* expression is more complex, since it is also affected by growth rate dependence on concentrations of regulatory factors, and further studies are required to understand *dnaK* expression under different growth conditions.

Our data show that 5′-end truncated *dnaK* mRNAs were dominant in infected eukaryotic cells and in 243 growth medium, as compared to 5′-end *dnaK* mRNAs from free bacterial grown in McCoy’s 5A medium. A likely explanation is that the abundance of 5′-end truncated transcripts results in a more rapid RNA turnover within the context of eukaryotic cell infection. This in turn would represent an advantageous nutrient rich environment for the parasitic life mode of mycoplasma.

We also show that the full-length protein is expressed, as demonstrated by cRT-PCR identification of complete 5′-end transcripts containing the ATG start codon and Western blot analysis. However, we also observed other truncated *dnaK* mRNA in infected eukaryotic cells, and the significance of these forms is not clear. Regarding the gene regulation, we found three putative *dnaK* internal promoters, and showed that few of the *dnaK* mRNAs identified by cRT-PCR had the promoters localized in the origin of the 5′-end truncated sequences, suggesting that some of the 5′-end truncated transcripts may be initiated from internal promoters. We could not rule out that *dnaK* transcription utilizes an alternative internal promoter/s and that therefore shorter forms of *dnaK* protein could be expressed. However, we did not observe any shorter protein in our Western blot assays. This could mean that either the shorter proteins are expressed at levels below detection, or that our monoclonal antibody is not able to detect them.

Overall, our data indicate that elevated expression of bacterial *dnaK* RNA and DnaK protein is present even when intracellular bacteria reach a replicative steady state of growth. Given the ability of bacteria to secrete DnaK [27,28], this would indicate that over time, the protein present in the intracellular compartments [24] could affect important cellular pathways. To this regard, in our previous paper we described the effect of DnaK in reducing activities of PARP1 and p53 [15,17,24,26]. Further studies are ongoing in our laboratory to identify other proteins bound to DnaK and the effect on their activity. Sequence analysis of *dnaK* mRNA transcripts highlighted the presence of defective transcripts with frequent internal single point mutations, suggesting that the high *M. fermentans* MF-I1 RNA turnover could be not only a result of the environmental adjustment, but also of a faulty transcription.

In conclusion, our study advances the understanding of transcriptional regulation of the essential *M. fermentans* MF-I1 *dnaK* gene during invasion of eukaryotic cells and reveals considerable complexity. Identification of the major bacterial and host components that govern the process of RNA turnover is required to further elucidate the mechanisms of M. *fermentans* survival and *dnaK* expression inside eukaryotic cells.

## Figures and Tables

**Table 1:** Invasion of HCT116 cells by Mycoplasma fermentans MF-I1.

Time Point	Internalized MF-I1 (CFU/10^6^ HCT116 Cells)	MF-I1 in Supernatant from HCT116 Culture (CFU/mL)	MF-I1 in McCoy’s 5A Medium Axenic Culture (CFU/mL)
Day 1	8242 ± 2224	8193 ± 3461	905 ± 75
Day 3	62,647 ± 3512	33,303 ± 3461	3568 ± 1275
Day 6	26,371 ± 596	336,665 ± 42,757	7874 ± 903

**Table 2:** Putative MF-I1 *dnaK* promoters.

*dnaK* Promoters	−35 Box	−10 Box
Pr. 1	(−173) TTGATT	(−153) ATTTAATTT
Pr. 2	(+197) TTGCAT	(+219) GGGTACAAA
Pr. 3	(+617) ATGAAA	(+638) TGCTAAAAT
Pr.4	(+1205) TTGAAA	(+1224) TGTTACAAT

In parenthesis is indicated the nucleotide distance of the box sequence from the MF-I1 *dnaK* ATG start codon.

**Table 3:** Nucleotide position due to mutations in *dnaK* transcripts from HCT116 infected with MF-I1 (H1 to H41), MF-I1 grown in axenic conditions in 243 medium (C1 to C40) and free bacterial grown in McCoy’s 5A medium (M1 to M40). Amino acid changes are indicated in the single point mutations.

CLONE	POINT MUTATIONS		INSERTIONS	DELETIONS
	**Nucleotide substitution**	**Amino acid substitution**		
H5	A1190G	Glu397Gly		
H6	C1578G	Asn526Lys		
H8	A756G	Leu255Leu		
H9	A1190G	Glu397Gly		
H10	A1190G	Glu397Gly		
H11	A1190G	Glu397Gly		
H12	G373T, T600C, A1236G	Ala125Ser, Gly200Gly, Lys412Lys		
H16	A1488G	Glu483Gly		
H19	A1512G	Ala504Ala		
H21				A1348 (Lys450)
H24	A1299G	Thr433Thr		
H28	T1304C, G1357A	Ile435Thr, Ala453Thr		
H32	G1321A	Gly441Arg		
H33	A1714G	Ser572Gly		
H35	G1471A, A1484G	Glu491Lys, Asn495Ser		
H39	A1211G	Asn404Thr		
H41	A1200G	Thr400Thr		
C2	A1368G, T1564C	Ile456Met, Val522Ala		
C9			790G (His264)	
C10	T648C	Ile216Ile		
C11	G4A, C1309T	Pro2Ser, Ala437Thr		
C13	C528G	Phe176Leu		
C15	T1263C	Ala421Ala		
C16	G1715A	Ser572Asn		
C20	C732T	Asp244Asp		
C24	A1075G, T1076C	Ile359Ala		
C26	A11G, T57C	Glu4Gly, Ala19Ala		
C34	A1503C	Lys501Asn		
C35	C761T, A1396G	Ser254Leu, Thr466Ala		
C37	A37G	Thr13Gly		
C40	C710T	Ala237Val		
M25	T1062G	Val354Val		
M26	T1070C	Arg390Arg		
M30	G632T	Trp211Leu		
M34	T764G, G765T	Leu259Leu, Val260Phe		
M35	C1298T	Thr433Ile		

## Data Availability

Data is contained within the article or supplementary material. The whole genome sequence *of Mycoplasma fermentans* strain MF-I1 has been deposited at NCBI GenBank under the accession number ATFG00000000.

## Supplementary Materials

The following are available online at https://www.mdpi.com/article/10.3390/ijms22083885/s1.

## Abbreviations

MF-I1	*Mycoplasma fermentans*-IHV-substrain 1
PCR	Polymerase Chain Reaction
PARP1	Poly (ADP-Ribose) Polymerase-1
USP10	Ubiquitin carboxyl-terminal hydrolase 10
CFU	Colony Forming Units
