# Supplementary material for: Analysis of DnaK Expression from a Strain of Mycoplasma fermentans in Infected HCT116 Human Colon Carcinoma Cells
Source: Int J Mol Sci. 2021 Apr 9;22(8):3885. doi: 10.3390/ijms22083885 (PMC8069837; doi:10.3390/ijms22083885)
Supplement: Supplementary file 1 [file ijms-22-03885-s001.zip › Suppl proof/Legend to Suppl. figures.docx]

**Fig. S1. Percentage of *dnaK* DNA copy number in different intracellular compartments at day 6. Upper part**: after six days in culture, DNA was collected from the different cellular compartments, and quantified by qPCR analysis according to the protocol described in Materials and Methods. After quantification, % copy number in each compartment was calculated. **Lower part**: Western Blot assay for specific proteins was performed to verify the correct intracellular compartment analyzed. Data represent the mean ± S.E. of samples run in triplicate and are representative of data from three different experiments. Cellular compartments are indicated as follows: C; Cytoplasm; M+O: Mitochondria + Organelles; N: Nucleus.

**Fig. S2. Schematic representation of circularized RT-PCR (cRT-PCR) protocol for analysis of mycoplasma *dnaK* mRNA.** The experimental design for the cRT-PCR RNA analysis is shown. At the top the target RNA is shown with the locations of the forward and reverse primers. In panel A the target primers were selected at the 5’ and 3’ end of the gene to identify the sequences of the 5’ and 3’ends of the target gene. In panel B the target primers were selected in the middle of the gene in order to determine the length of the transcripts. In the circularization step the 5’ and 3’ ends were linked by T4 RNA ligase and the RNA subjected to gene-specific reverse transcription. The first PCR was performed using the F1 and R2 primers. The second PCR used the internal F2 and R2 primers. Products from the second PCR were subsequently cloned and sequenced, as described under Material and Methods.

**Fig. S3. Multiple sequence alignment of *dnaK* mRNAs.** Shown is a sequence alignment of 41

*dnaK* mRNA sequences from HCT116 cells infected with MF-I1 (H1-H41), 40 from the MF-I1

axenic culture in 243 medium (C1-C40) and 40 from the MF-I1 axenic culture in McCoy's 5A

medium (M1-M40) that was generated with Muscle (Edgar, 2004) and edited using Jalview

(Waterhouse, 2009). The consensus included a sequence logo and the sequences of *Mycoplasma*

*fermentans* MF-I1 and *Mycoplasma fermentans* JER. This alignment corresponds to the 48558-

48376 positions in the reference genome of *Mycoplasma fermentans* MF-I1 (ATFG00000000)

including the complete *dnaK* gene with the ATG initiation codon (position 48569) marked as 51

in the figure. This alignment also corresponds to the 104955-106800 positions in the reference

genome of *Mycoplasma fermentans* JER (CP001995) including the complete *dnaK* gene with the

ATG initiation codon (position 104993) marked as 51. Most mutations were individual with the

exception of a single guanine substitution in position +1190 nucleotide after the ATG start codon

that was shared among 4 clones.
