# Supplementary material for: Analysis of DnaK Expression from a Strain of Mycoplasma fermentans in Infected HCT116 Human Colon Carcinoma Cells
Source: Int J Mol Sci. 2021 Apr 9;22(8):3885. doi: 10.3390/ijms22083885 (PMC8069837; doi:10.3390/ijms22083885)
Supplement: Supplementary file 1 [file ijms-22-03885-s001.zip › Suppl proof/Table S1.pdf]

Table S1. List of primers for determining 5’ and 3’ -ends of *dnaK, rnhB1* and *msg1* mRNAs.

gene	Primer 5’-3’	nucleotide distance from origin of gene
rnhB1	**R1** CTGCAGCACAACAAAGTGGA	+90 (47639-47658)
	**F1** GTGAATTCTTCAAAAATCATCCT	+617 (48166-48188)
	**R2** ATCGCCCACTCCTACTTCG	+63 (47612-47630)
	**F2** CTATTAATTTGTTTGCAACAGATTTTG	710 (48259-48285)
dnaK	**R1** CGATTGGTGTACCACCATCAAC	+61 (48629-48650)
	**F1** GAATATGACCAACTTCGTTTAACA	+1656 (50225-50248)
	**R2** ACAGCTGAGTTAGTTGTACCT	+33 (48601-48621)
	**F2** AATGAGCAATGCAGACTCATCA	+1700 (50278-50299)
mgs1	**R1** AACTGCTGCTGAGCTTTTTCC	+139 (50653-50673)
	**F1** TCACTGGGTAAAACAGGAGTA	+1086 (51600-51620)
	**R2** GAGACAACATGCTTTTGGCC	+52 (50566-50585)
	**F2** GAACCAGATAATAATGATTCAC	+1141 (51655-51676)
