# Supplementary material for: Analysis of DnaK Expression from a Strain of Mycoplasma fermentans in Infected HCT116 Human Colon Carcinoma Cells
Source: Int J Mol Sci. 2021 Apr 9;22(8):3885. doi: 10.3390/ijms22083885 (PMC8069837; doi:10.3390/ijms22083885)
Supplement: Supplementary file 1 [file ijms-22-03885-s001.zip › Suppl proof/Table S2.pdf]

Table S2. List of primers for determining *dnaK* mRNA length.

gene	Primer 5'-3'	nucleotide distance from origin of gene
dnaK	**RL1** TGGTCTACGGCAACGTTCAA	+875 (49425-49444)
	**FL1** AGCTAAACTCAAGATTTCAGAC	+897 (49465-49486)
	**RL2** CGTTAATAGGTTCATGATTGTC	+784 (49352-49373)
	**FL2** ATGGGTGCTGCAATTCAAGG	+1036 (49604-49623)
