# Supplementary material for: Analysis of DnaK Expression from a Strain of Mycoplasma fermentans in Infected HCT116 Human Colon Carcinoma Cells
Source: Int J Mol Sci. 2021 Apr 9;22(8):3885. doi: 10.3390/ijms22083885 (PMC8069837; doi:10.3390/ijms22083885)
Supplement: Supplementary file 1 [file ijms-22-03885-s001.zip › Suppl proof/Tables S3-S7.pdf]

Table S3A. *DnaK* 5’ and 3’ -end sequences from *Mycoplasma fermentans* axenic culture in 243 medium.

CLONE	GENE	SEQUENCE BEFORE GENE START	SEQUENCE AFTER GENE STOP
D1	*DnaK*	MISSING	**TAG**TTAATTTAAAAATAAAAAAGTGCCAAATGCTGGTACTTTTTTA
D2-D3	*DnaK*	MISSING	**TAG**TTAATTTAAAAATAAAAAAGTGCCAAATGCTGGTACTTTTTTTAT
D4, D7	*DnaK*	MISSING	**TAG**TTAATTTAAAAATAAAAAAGTGCCAAATGCTGGTACTTTTTTTATTT
D5, D8, D10-D11	*DnaK*	MISSING	**TAG**TTAATTTAAAAATAAAAAAGTGCCAAATGCTGGTACTT
D6	*DnaK*	ATATACTAAAACGTATTTGAAAGGAAAATT**ATG**	**TAG**TTAATTTAAAAATAAAAAAGT
D9	*DnaK*	MISSING	**TAG**TTAATTTAAAAA
D12	*DnaK*	MISSING	**TAG**TTAATTTAAAAATAAAAAAGTGCCAAATGCTGGTA

Table S3B. *DnaK* 5’ and 3’ -end sequences from HCT116 cells infected with *Mycoplasma fermentans.*

CLONE	GENE	SEQUENCE BEFORE GENE START	SEQUENCE AFTER GENE STOP
D1	*DnaK*	AAATGCTGGTACTTTTTTATATATACTAAAACGTATTTGAAAGGAAAATT**ATG**	**TAG**TTAATTTAAAAATAAAAAAGTGCCAAATGCTGGTACTTTTTTTAT
D2	*DnaK*	AAATGCTGGTACTTTTTTATATATACTAAAACGTATTTGAAAGGAAAATT**ATG**	**TAG**TTAATTTAAAAATAAAAAAGTGCCAAATGCTGGCACTTTTTTA
D3	*DnaK*	MISSING	**TAG**TTAATTTAAAAATAAAAAAGTGCCAAATGCTGGCACTTTTTTA
D5	*DnaK*	TTTTTATATATACTAAAACGTATTTGAAAGGAAAATT**ATG**	**TAG**TTAATTTAAAAATAAAAAAGTGCCAAGTGCTGGTACTTTTTTTAT
D6	*DnaK*	TATATACTAAAACGTATTTGAAAGGAAAATT**ATG**	**TAG**TTAATTTAAAAATAAAAAAGTGCCAAATGCTGGTACTTTTTTTAT
D7	*DnaK*	TATATACTAAAACGTATTTGAAAGGAAAATT**ATG**	**TAG**
D8	*DnaK*	ATATACTAAAACGTATTTGAAAGGAAAATT**ATG**	**TAG**TTAATTTAAAAATAAAAAAGTGCCAAATGCTGGTACTTTTTTA
D9-12	*DnaK*	pGEM EMPTY VECTOR	

Table S4A. *RnhB1* 5’ -end sequences from *Mycoplasma fermentans* axenic culture in 243 medium.

CLONE	SEQUENCE
R1	AGCAGATTTGGAAGACTAATT**ATG**
R2	TCTGTAGCTGTTGGTAATGCACCAGATGCAGTTAAAAAATATGCAACAATCAAATTAAAGAAAACAAACCGCCAGGGAGCAGTTGGTGAATACATCAAT<br>AAATTCCTTGATAATCCTGAAAAAGAAATAGAAAAAAGTAATAAAGTTATGGCTAAATTAATTAAACGTCGTGACGAAGAAGCAGATTTGGAAGACTAATT**ATG**
R3	TCGTGACGAAGAAGCAGATTTGGAAGACTAATT**ATG**
R4	TTAAACGTCGTGACGAAGAAGCAGATTTGGAAGACTAATT**ATG**
R5	AAATATGCAACAATCAAATTAAAGAAAACAAACCGCCAGGGAGCAGTTGGTGAATACATCAATAAATTCCTTGATAATCCTGAAAAAGAAATAGAAAAAAGTAATAAAG<br>TTATGGCTAAATTAATTAAACGTCGTGACGAAGAAGCAGATTTGGAAGACTAATT**ATG**
R6	GCAGATTTGGAAGACTAATT**ATG**
R7	TATGCAACAATCAAATTAAAGAAAACAAACCGCCAGGGAGCAGTTGGTGAATACATCAATAAATTCCTTGATAATCCTGAAAAAGAAATAGAAAAAAGTAATAAAGTTA<br>TGGCTAAATTAATTAAACGTCGTGACGAAGAAGCAGATTTGGAAGACTAATT**ATG**
R8	TGACGAAGAAGCAGATTTGGAAGACTAATT**ATG**
R10	AGGGAGCAGTTGGTGAATACATCA<br>ATAAATTCCTTGATAATCCTGAAAAAGAAATAGAAAAAAGTAATAAAGTTATGGCTAAATTAATTAAACGTCGTGACGAAGAAGCAGATTTGGAAGACTAATT**ATG**
R11	ATTCCTTGATAATCCTGAAAAAGAAATAGAAAAAAGTAATAAAGTTATGGCTAAATTAAT TAAACGTCGTGACGAAGAAGCAGATTTGGAAGACTAATT**ATG**

Table S4B. *RnhB1* 5’ -end sequences from HCT116 cells infected with *Mycoplasma fermentans*.

CLONE	SEQUENCE
R1	ACTTTCATTATTAGCTAGATACTATGGAATTGATTTAGAACACACTGTAGCATTTGGTGATGGTTTCAACGATGTGCCTATGTTTA<br>AAGTTGCTAATATTTCTGTAGCTGTTGGTAATGCACCAGATGCAGTTAAAAAATATGCAACAATCAAATTAAAGAAAACAAACCGCCAGGGAGCAGTTGGTGAATACATCA<br>ATAAATTCCTTGATAATCCTGAAAAAGAAATAGAAAAAGTAATAAAGTTATGGCTAAATTAATTAAACGTCGTGACGAAGAAGCAGATTTGGAAGACTAATT**ATG**
R2	AGCAGATTTGGAAGACTAATT**ATG**
R3	AAAATATGCAACAATCAAATTAAAGAAAACAAACCGCCAGGGAGCAGTTGGTGAATACAT<br>CAATAAATTCCTTGATAATCCTGAAAAAGAAATAGAAAAAAGTAATAAAGTTATGGCTAAATTAATTAAACGTCGTGACGAAGAAGCAGATTTGGAAGACTAATT**ATG**
R4	AGTTAAAAAATATGCAACAATCAAATTAAAGAAAACAAACCGCCAGGGAGCAGTTGGTGAATAC<br>ATCAATAAATTCCTTGATAATCCTGAAAAAGAAATAGAAAAAAGTAATAAAGTTATGGCTAAATTAATTAAACGTCGTGACGAAGAAGCAGATTTGGAAGACTAATT**ATG**
R5	TTATGGCTAAATTAATTAAACGTCGTGACGAAGAAGCAGATTTGGAAGACTAATT**ATG**
R7	TGACGAAGAAGCAGATTTGGAAGACTAATT**ATG**
R8	TTATGGCTAAATTAATTAAACGTCGTGACGAAGAAGCAGATTTGGAAGACTAATT**ATG**
R9	TCGTGACGAAGAAGCAGATTTGGAAGACTAATT**ATG**
R10	MISSING
R11	GAAGCAGATTTGGAAGACTAATT**ATG**
R12	AATTCCTTGATAATCCTGAAAAAGAAATAGAAAAAAGTAATAAAGTTATGGCTAAATTAATTAAACGTCGTGACGAAGAAGCAGATTTGGAAGACTAATT**ATG**

Table S4C. *RnhB1* 5’ -end sequences from *Mycoplasma fermentans* axenic culture in McCoy's 5A medium.

CLONE	SEQUENCE
R1, 7, 8, 9	TTATGGCTAAATTAATTAAACGTCGTGACGAAGAAGCAGATTTGGAAGACTAATT**ATG**
R2, 3,4, 5, 6, 10, 11, 12	GACGAAGAAGCAGATTTGGAAGACTAATT**ATG**

Table S5A. *RnhB1* 3’ -end sequences from *Mycoplasma fermentans* axenic culture, in 243 medium.

CLONE	SEQUENCE
R1, 5, 10	**TAA**TTAAATTCAACTTTTCTAAAGCAAAAAATAAAAAAATATTATCATTTTTATGCAAATTAGAGACGTGCGGATCTAATTATTTTTTTA
R2, 7	**TAA**TTAAATTCAACTTTTCTAAAGCAAAAAATAAAAAAATATTATCATTTTTATGCAAATTAGAGACGTGCGGATCTAATTATTTTTTTAT
R3	**TAA**TTAAATTCAACTTTTCTAAAGCAAAAAATAAAAAAATATTATCATTTTTATGCAAATTAGAGACGTGCGGATCTAATTATTTT
R4, 11	**TAA**TTAAATTCAACTTTTCTAAAGCAAAAAATAAAAAAATATTATCATTTTTATGCAAATTAGAGACGTGCGGATCTAATTATTTTTT
R6, 8	**TAA**TTAAATTCAACTTTTCTAAAGCAAAAAATAAAAAAATATTATCATTTTTATGCAAATTAGAGACGTGCGGATCTAATTATTTTTTT

Table S5B. *RnhB1* 3’ -end sequences from HCT116 cells infected with *Mycoplasma fermentans.*

CLONE	SEQUENCE
R1, 3, 5, 7, 8, 9, 11	**TAA**TTAAATTCAACTTTTCTAAAGCAAAAAATAAAAAAATATTATCATTTTTATGCAAATTAGAGACGTGCGGATCTAATTATTTTTT
R2	**TAA**TTAAATTCAACTTTTCTAAAGCAAAAAATAAAAAAAATATTATCATTTTTATGCAAATTAGAGACGTGCGGATCTAATTATTTTTTTA
R4	**TAA**TTAAATTCAACTTTTCTAAAGCAAAAAATAAAAAAATATTATCATTTTTATGCAAATTAGAGACGTGCGGATCTAATTATTTTT
R10	**TAA**TTAAATTCAACTTTTCTAAAGCAAAAAATAAAAAAATATTATCATTTTTATGCAAATTAGAGACGTGCGGATCTAATTAT
R12	**TAA**TTAAATTCAACTTTTCTAAAGCAAAAAATAAAAAAAATATTATCATTTTTATGCAAATTAGAGACGTGCGGATCTAATTATTTTTTTATTC<br>CTT

Table S5C. *RnhB1* 3’ -end sequences from *Mycoplasma fermentans* axenic culture in McCoy's 5A medium.

CLONE	SEQUENCE
R1, 7, 8, 9	**TAA**TTAAATTCAACTTTTCTAAAGCAAAAAATAAAAAAATATTATCATTTTTATGCAAATTAGAGACGTGCGGATCTAATTATTTTTTT
R2, 3, 4, 5, 6, 10, 11, 12	MISSING

Table S6. *Mgs1* 5’ -end sequences from *Mycoplasma fermentans* axenic culture in 243 medium, HCT116 cells infected with *Mycoplasma fermentans* and *Mycoplasma fermentans* axenic culture in McCoy's 5A medium

Mycoplasma axenic culture, 243 medium		HCT116 cells infected with *Mycoplasma*		*Mycoplasma axenic* culture, McCoy's 5A medium	
**CLONE**	**SEQUENCE**	**CLONE**	**SEQUENCE**	**CLONE**	**SEQUENCE**
A1	**ATG**	A1	**MISSING**	A1	**ATG**
A2	**ATG**	A2	**ATG**	A2	**ATG**
A3	**ATG**	A3	**ATG**	A3	**ATG**
A4	**ATG**			A4	**ATG**
A5	**MISSING**	A5	**ATG**	A5	**ATG**
A6	**MISSING**	A6	**ATG**	A6	**ATG**
A7	**ATG**	A7	**ATG**	A7	**ATG**
A8	**ATG**	A8	**MISSING**	A8	**ATG**
A9	**MISSING**	A9	**ATG**	A9	**MISSING**
A10	**MISSING**	A10	**ATG**	A10	**ATG**
A11	**ATG**	A11	**ATG**	A11	**ATG**
A12	**ATG**	A12	**ATG**	A12	**ATG**

Table S7A. *Mgs1* 3’ -end sequences from *Mycoplasma fermentans* axenic culture, in 243 medium.

**CLONE**	**SEQUENCE**
A1, 2, 3, 4, 7	**TAA**CATGAATGATATTAAATGAGAAGAAATCAAAACATTTGAAGATTTAAAATTAGCTAAAAGCAAGGTATATTCAAAGGA
A5, 6, 9, 10	**TAA**CATGAATGATATTAAATGAGAAGAAATCAAAACATTTGAAGATTTAAAATTAGCTAAAAGCAAA
A8, 11, 12	**TAA**CATGAATGATATTAAATGAGAAGAAATCAAAACATTTGAAGATTTAAAATTAGCTAAAAGCAAAGTATATTCAAAGGACTCTGAAATAGTTCAATTACAAACA AAAATTAGAACAGCTTCGCCT

Table S7B *Mgs1* 3’ -end sequences from HCT116 cells infected with *Mycoplasma fermentans*.

CLONE	SEQUENCE
A1	**TAA**CATGAATGATATTAAATGAGAAGAAATCAAAACATTTGAAGATTTAAAATTAGCTAAAAGCAAAGTATATTCAAAGGACTCTGAAATAGTTCAATTACAAACA<br>AAAATTAGAACAGCTTCGCCTGAAGAGAAAAAAGAAATAGGTCAAAAAATTGCAACTTTAAGAAACTTTTATGAAGAAAAATTCAAAGAAATTGAATTTAAAATTG<br>AAGCCGATAAAATCAAAAAAATAATTGAAAGCCAATATGTTGATGTAACTAAACCAGTTGACAATCCTGGTTCATTGCACCCAATTACAATAGTAGAAAATAGACTT<br>AGAGATTGATTTATTCAAAACGGATATTTTGAATCAAATGAAAGTGAAATTGTTAGCGATGTTTATAATTTTCAAAAATTAAACATTCCTGAAGATCACCCTGCTCGT<br>GCTATGCACGATTCATTATATTTAAATCCAACAACTCTTTTAAGAACACACAACACTGGAATTAGTGCACTTGAACTTGAAAGAAATGCAAACAAAGAAATGAATAA<br>TTTCGCTATTGGAAAAGTTTATCGTAATGATGAAGATGATGCAACTCACTCACATCAATTTACACAATTAGACTTTGTCAGTGTTGGCAAAAGTGTAAGCTTCAACAA<br>TTTAATTTGAACACTAAAATCACTTCTTTCTTATGTATTTGAAGAAGAAGTTGAACTAAGACTTAGACCAAGTTATTTCCCTTTTACTGAACCAAGTGTTGAAGTCGAC<br>GTTTATTATAAAAATGGTTGAATCGAAGTTTTAGGTGCTGGTATGCTTCATCCAAATGTTATGGAACTTGCAGGTTATGATTCAAAAGAATTTAACGGTTTTGCTGCA<br>GGCATTGGAGTTGAACGTTTGACAATGATTAAATATAACATCAAGGATGTTAGAGAATTTTATAAAAATGACTTGAGAACTTTAAATCAATTTAACTATGAAGAATA<br>GCTTCCAAATATTTTTAAAACAAGAAGAATCTAAAAACTATTATCAAGAATTAATAAAAAAAGTTACTCAAGAAGAACAAAAACACAATGTTTTTCCACCTAAAGAA<br>TTACGTTTTGATGCTTTAAATTATTTTGAAGCAGAAGAGACTAAATTAATAATAATTGGACAAGATCCTTATTATTTAAAAAACCAAGCAGATGGTTTAGCTTTTAGT<br>ACTCAAGGCAATATTTGTCCTAAAAGTTTAAACAATATTTTTAAAGAATTAAAGAAAGATTATTCTGAAACAAACATTGAAACTTATAGCTTAAAAAGTTGAGCAAA<br>ACAAGGAGTTTTATTAATTAATACTTGTTTAAGTGT TAATGAAAATCAACCATTAAGCCATAAGAATTTTGGCTGA
A2	**TAA**CATGAATGATATTAAATGAGAAGAAATCAAAACATTTGAAGATTTAAAATTAGCTAAAAGCAAAGTATATTCAAAGGACTCTGAAATAGTTCAATTACAAACA<br>AAAATTAGAACAGCTTCGCCTGAAGAGAAAAAAGAAATAGG
A3	**TAA**CATGAATGATATTAAATGAGAAGAAATCAAAACATTTGAAGATTTAAAATTAGCTAAAAGCAAAGTATATTCA
A5, 6, 7	**TAA**CATGAATGATATTAAATGAGAAGAAATCAAAACATTTGAAGAT TTAAAAT
A8	**TAA**CATGAATGATATTAAATGAGAAGAAATCAAAACATTTGAAGATTTAAAATTAGCTAAAAGCAAAGTATATTCAAAGGACTCTGAAATAGTTCAATTACAAACA<br>AAAATTAGAACAGCTTCGCCTGAAGAGAAAAAAGAAATAGGTCAAAAAATTGCAACTTTAAGAAACTTTTATGAAGAAAAATTCAAAGAAATTGAATTTAAAATTG<br>AAGCCGATAAAATCAAAAAAATAATTGAAAGCCAATATGTTGATGTAACTAAACCAGTTGACAATCCTGGTTCATTGCACCCAATTACAATAGTAGAAAATAGACTT<br>AGAGATTGATTTATTCAAAACGGATATTTTGAATCAAATGAAAGTGAAATTGTTAGCGATGTTTATAATTTTCAAAAATTAAACATT CCTGAAGATCACCCTGCTC
A9	**TAA**CATGAATGATATTAAATGAGAAGAAATCAAAACATTTGAAGATTTAAAATTAGCTAAAAGCAAAGTATATTCAAAGGACTCTGAAATAGTTCAATTACAAACA<br>AAAATTAGAACAGCTTCGCCTGAAGAGAAAAAAGAAATAGGTCAAAAAATTGCAACTTTAAGAAA
A10	**TAA**CATGAATGATATTAAATGAGAAGAAATCAAAACATTTGAAGATTTAAAATTAGCTAAAAGCAAAGTATATTCA
A11	**TAA**CATGAATGATATTAAATGAGAAGAAATCAAAACATTTGAAGATTTAAAATTAGCTAAAAGCAAAGTATATTCAAAGGACTCTGAAATAGT
A12	**TAA**CATGAATGATATTAAATGAGAAGAAATCAAAACATTTGAAGATTTAAAATTAGCTAAAAGCAAAGTATATTCAAAGGACTCTGAAATAGTTCAATTACAAACA<br>AAAATTAGAACAGCTTCGCCTGAAGAGAAAAAA
A12	**TAA**CATGAATGATATTAAATGAGAAGAAATCAAAACATTTGAAGATTTAAAATT

Table S7C. *Mgs1* 3’ -end sequences from *Mycoplasma fermentans* axenic culture in McCoy's 5A medium.

CLONE	SEQUENCE
A1, 2, 3, 4, 6, 7, 10, 11, 12	**MISSING**
A5	**TAA**CATGAATGATATTAAATGAGAAGAAATCAAAACATTTGAAGATTTAAAATTAGCTAAAAGCAAAGTATATTCAAAGGACTCT
A9	**TAA**CATGAATGATATTAAATGAGAAGAAATCAAAACATTTGAAGATTTAAAATTAGCTAAAAGCAAAGTATATTCAAA
