# Supplementary figures and images for: Analysis of DnaK Expression from a Strain of Mycoplasma fermentans in Infected HCT116 Human Colon Carcinoma Cells
Source: Int J Mol Sci. 2021 Apr 9;22(8):3885. doi: 10.3390/ijms22083885 (PMC8069837; doi:10.3390/ijms22083885)

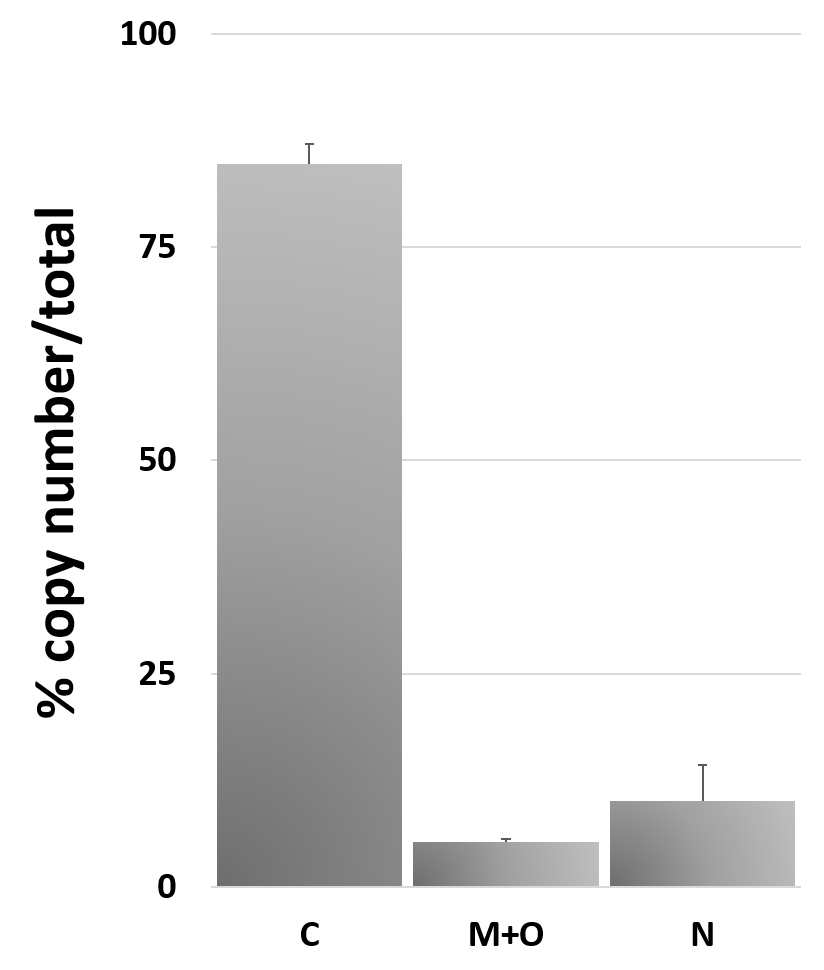

Supplement: Supplementary file 1 [file ijms-22-03885-s001.zip › Suppl proof/Fig.S1.png]

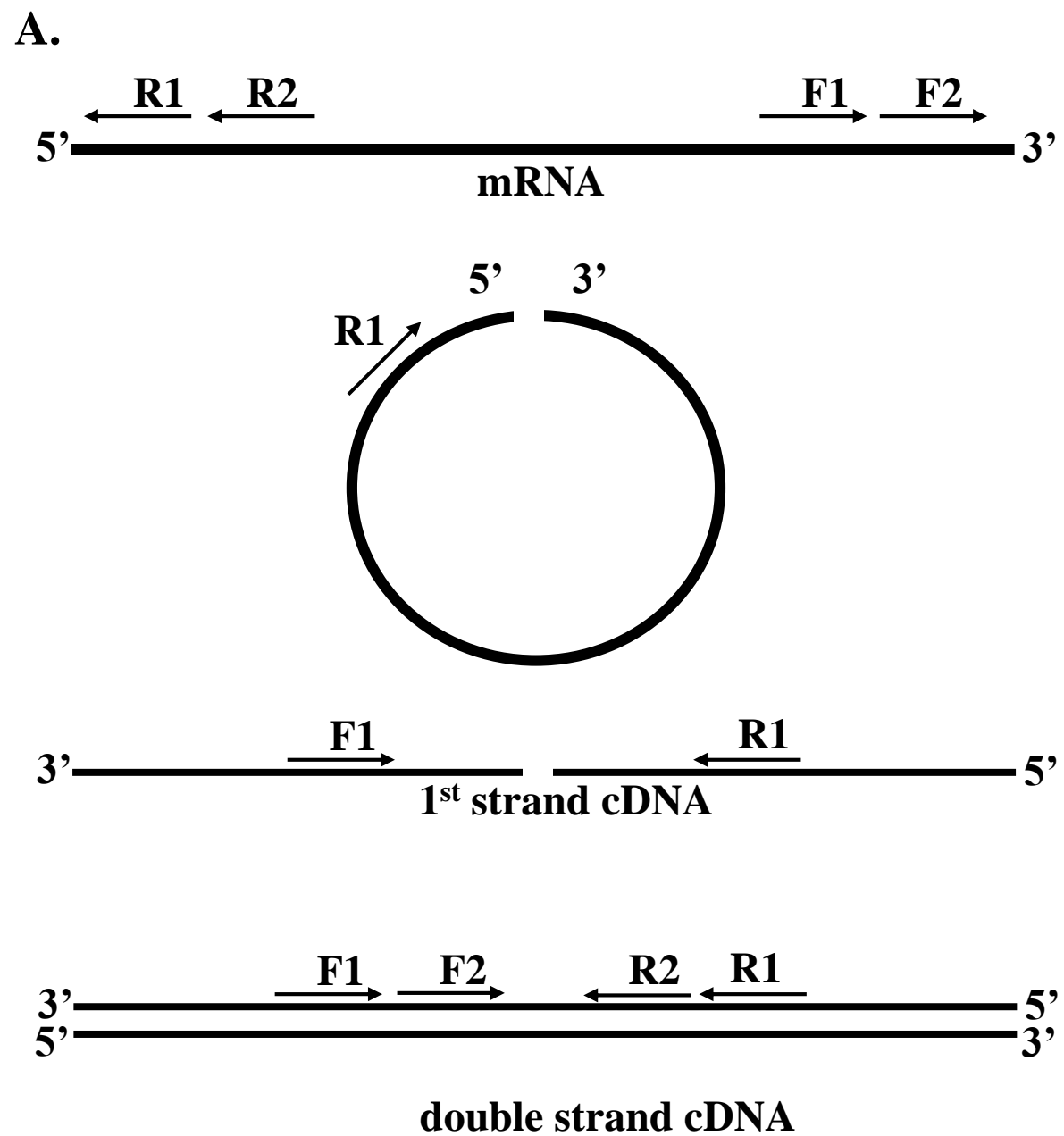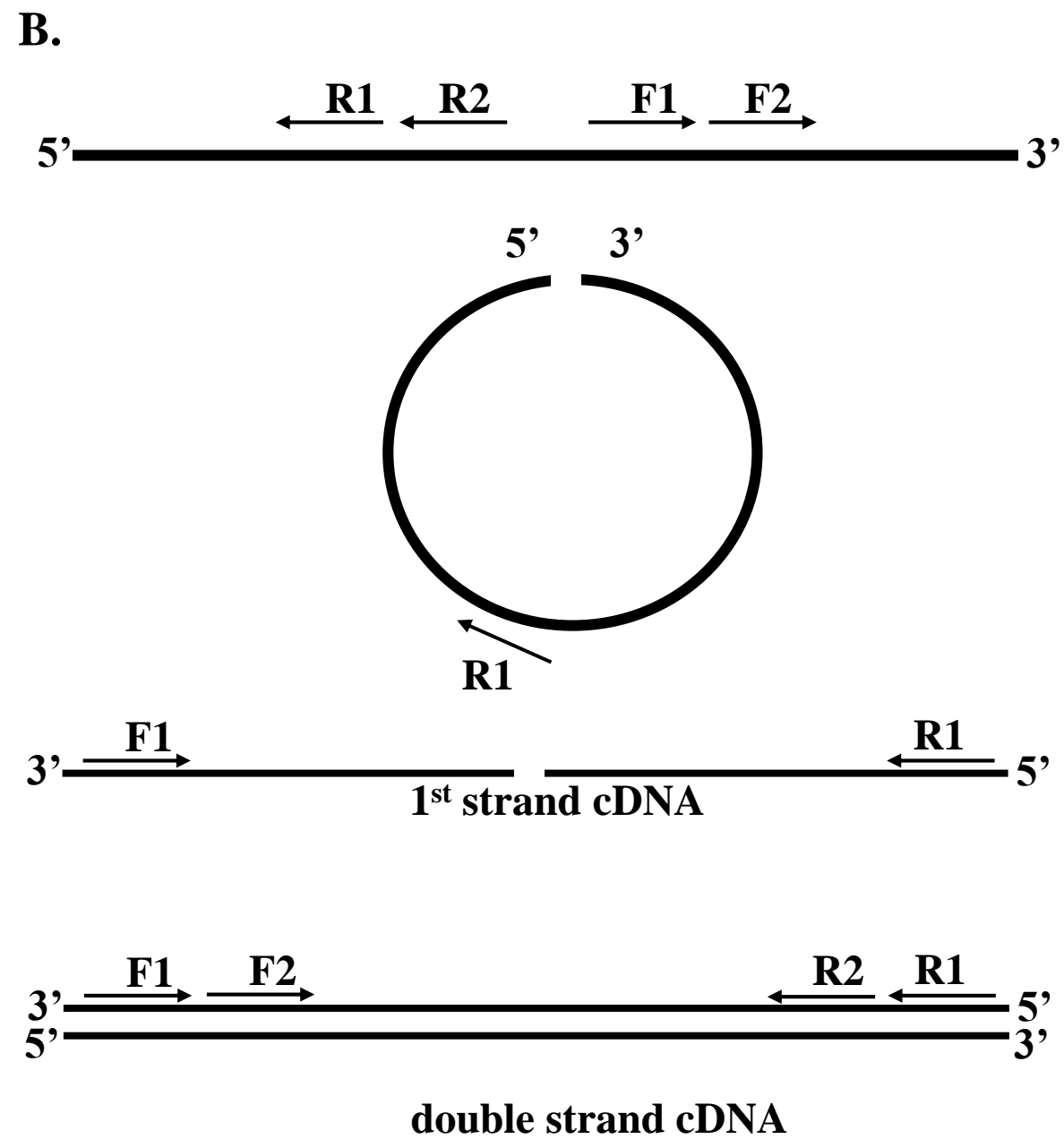

Supplement: Supplementary file 1 [file ijms-22-03885-s001.zip › Suppl proof/Fig.S2.pdf]

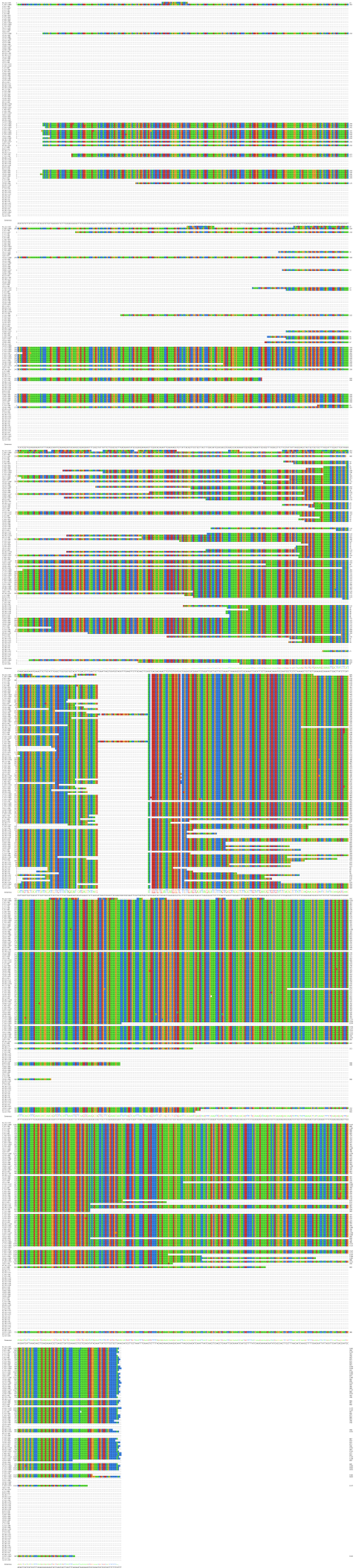

Supplement: Supplementary file 1 [file ijms-22-03885-s001.zip › Suppl proof/FIG.S3.pdf]
